# Wireless Heart Rate Variability in Assessing Community COVID-19

**DOI:** 10.3389/fnins.2021.564159

**Published:** 2021-06-08

**Authors:** Robert L. Drury, Marc Jarczok, Andrew Owens, Julian F. Thayer

**Affiliations:** ^1^Canary Systems, Bainbridge Island, WA, United States; ^2^Clinic for Psychosomatic Medicine and Psychotherapy, University Clinic Ulm, Ulm, Germany; ^3^Department of Old Age Psychiatry, Institute of Psychiatry, Psychology & Neuroscience, King's College London, London, United Kingdom; ^4^Psychological Sciences Faculty, University of California, Irvine, Irvine, CA, United States

**Keywords:** heart rate variability, COVID-19 pandemic, community prevalence, networked hardware/software systems, digital epidemiology, Internet of Healthy Things (IoHT)

## Introduction

According to the Johns Hopkins Coronavirus Resources Center, the number of confirmed COVID-19 cases exceeds 170,558,922 worldwide today with more than 3,546,881 fatalities (2021). The pandemic's massive health and well-being issues have already impacted the lives of millions globally, including spikes in mortality and morbidity. Many nations were unprepared for these significant consequences, revealing the critical need for standard public health principles of population assessment, intervention, and treatment. This editorial will address an innovative use of Heart Rate Variability (HRV), which is a non-invasive, inexpensive, and sensitive measure of inflammatory processes and immunomodulation (Kovatchev et al., 2003; Ahmad et al., 2009; Leitzke et al., 2020; Owens, 2020), among other health and well-being parameters. Specifically, the vagus nerve maintains tonic inhibitory control of proinflammatory cytokines via acetylcholine release into the reticuloendothelial system (e.g., spleen, gastrointestinal tract, heart, liver), mediating the inflammatory reflex through the cholinergic anti-inflammatory pathway (Dantzer and Kelley, 2007). HRV has been described in this special HRV Horizons 2030 Frontiers series as follows: “HRV offers insights into humoral, neural, and neurovisceral processes in health and disorders of brain, body, and behavior but has yet to be fully potentiated in the digital age” (Owens, 2020). Building on a growing body of HRV data (Rangon et al., 2020; Whitelaw et al., 2020; Hirten et al., 2021), we propose use of a wearable high fidelity Oura sensor ring (https://ouraring.com/blog/category/research-validation/) to acquire HRV, in addition to other physiological indicators, to track both pre-illness longitudinal baseline and an ongoing longitudinal Community assessment of those indicators associated with COVID-19 using algorithmic analysis and actionable feedback. While various aspects of this proposal have been used by investigators producing promising results at UC San Francisco (Smarr et al., 2020), UC San Diego/Scripps Research (Whitelaw et al., 2020), Stanford School of Medicine (Perez et al., 2019), Mt. Sinai's Icahn School of Medicine (Hirten et al., 2021), and others (Chung, 2020; Hasty et al., 2020), we propose a synthetic approach that incorporates the advantages of the most promising, actionable and practical elements to elucidate how HRV can act as a predictor of COVID19 infection. The use of longitudinal HRV data acquired by a personal device, transferred by smart phone application and analyzed by high throughput cloud-based machine learning algorithm represents an innovative, inexpensive, easily deployable, and scalable method for both individual use for health behavior maintenance and for communication and decision support with clinical and public health professionals in communities and larger jurisdictions.

HRV research has produced extensive literature, with a recent PubMed search of the term “heart rate variability” producing more than 50,000 citations (Malik and Camm, 2004; Shaffer and Ginsberg, 2019), with rapidly evolving neuroscientific HRV studies (Holzman and Bridgett, 2017). Major theoretical contributions have been made by Porges' Polyvagal Theory (Porges, 2011); Grossman's biobehavioral studies of cardiac vagal tone (Grossman and Taylor, 2007); Owens, Critchley and associates studies of HRV as a remote digital biomarker (Owens et al., 2018); and Thayer's neurovisceral integration approach (Thayer and Lane, 2020), all show the important role of HRV as a physiological indicator of inflammatory and immune system activity. Briefly, HRV is the instantaneous variation in the inter-beat interval (IBI) of the electrocardiogram. HRVs relation to many disease states and human psychophysiological functions has been studied extensively. Perhaps counterintuitively, greater variability in IBI, measured as the time between adjacent R to R peaks in the ECG, is positively correlated to fewer and/or lesser negative health or well-being consequences in many diseases and conditions. These constant allostatic variations can be seen as analogous to the over 22,000 course corrections necessary for Apollo 8 to land on the moon (McEwen, 2017). Recent reviews have described the wide variety of applications of HRV in both medical and psychosocial settings (Drury et al., 2019). In particular, the Thayer group showed that HRV is related to inflammatory processes in humans (Williams et al., 2019; Jarczok et al., 2021) and identified an HRV related cholinergic anti-inflammatory pathway (Thayer and Fischer, 2009). Investigators have explored the use of HRV in medical conditions, including infectious and immune related disorders, in both human and animal studies showing various HRV parameters to be related to infection and immune system function (Fairchild, 2013; Herry et al., 2016; Pavlov and Tracey, 2019; Pavlov et al., 2020).

Based on this body of basic and applied HRV research, we wish to urgently propose using HRV monitoring as an element of a larger framework of truly personalized health (Drury, 2019; Hood et al., 2019). HRV screening, analysis and feedback can be applied immediately to the present COVID-19 pandemic. A recent report by Jarczok et al. (2019) has presented proof of concept of HRV as a marker of health risks in human adults. We propose applying the Jarczok et al. method to IBI data obtained from personal devices such as the Oura ring, Apple Watch, Fitbit, and the Polar strap, among others, facilitating scalability, accessibility, economy and high fidelity data acquisition. The Apple Heart Study conducted by Stanford University's School of Medicine demonstrated the feasibility of using wearable technology, specifically the Apple Watch, to examine cardiovascular data for atrial fibrillation. They point out “this is just the beginning, as this study opens the door to further research into wearable technologies and how they might be used to prevent disease before it strikes—a key goal of precision health” (Perez et al., 2019). Validating this point is the report of Hirten et al. (2021) which used Apple Watches to acquire and process HRV and circadian HRV analysis to detect COVID-19 symptoms up to 7 days before they emerge clinically. We propose an iP4 model of healthcare, an approach that Integrates: Personalized, Prescriptive, Participatory, and Preventive variables in modeling human health and function (Drury, 2019; Hood et al., 2019) and is similar to the precision medicine concept recently advocated by NIH Director Collins (Denny and Collins, 2021).

An HRV measure sensitive to the inflammatory processes in viral infection is the root mean square of successive differences between normal heart beats (RMSSD). Jarczok et al. (2019) developed an algorithm that converts IBI to RMSSD, which is then associated with appropriate risk factor values. We propose an developing a smart phone app containing the algorithm made widely available, which would obtain IBI from a suitable personal device (e.g., Oura Ring, Apple Watch, etc.). Data is analyzed on the app, allowing an assessment of a HRV-derived COVID-19 risk factor in addition to other physiological measures, and subjective symptom and demographic data assessment using the COVID-19 Symptom Tracker (Chan et al., 2020). This algorithm would prompt appropriate health behaviors, including judiciously seeking medical help if the risk threshold is reached. With informed consent, the information would be encrypted and transferred to the appropriate local health authority for tracking community COVID-19 prevalence. This approach could be rapidly deployed. As recently as 2015, 64% of the overall US population and 82% of those aged 18-49 own app-enabled smart phones (Smith et al., 2015). The most recent data available from Statistica^1^ on smart phone deployment shows there are 3.8 billion app enabled smart phones worldwide (2020). Apple alone reports more than 22 million Apple Watches shipments in 2018, and several other smart watches and devices with IBI capability exist, such as Elite HRV CorSense, Fitbit, Polar straps, and Garmin monitors.

The current problems with widespread assessment of COVID-19 prevalence are urgent with high case incidence, intensive care unit occupancy and case fatalities. The US life span has declined by 1 year in the first 6 months of 2020, the biggest decline since World War 2 (Arias et al., 2021). We have proposed here an integrated systems approach to acquiring relevant individual data, analyzing it algorithmically, and producing health prompts based on those analyses. Our algorithm will be refined by including HRV and other physiological parameters such as temperature, respiration, SPO2, and activity level. Optimal complementary HRV parameters will also be explored by the use of sophisticated data analytic platforms such as Kubios (Tarvainen et al., 2014). This would allow community monitoring of likely COVID-19 status, which would be subject to further investigation and intervention by public health authorities as indicated. Participants would receive data driven health information including prompts for appropriate health behavior. Beyond this critical application, proactive use of the proposed approach would also be relevant for public health and clinical medical application. Now that most responsible national, state, and local governments have recognized the threat posed by the COVID-19 pandemic, the HRV monitoring proposed here can also be incorporated into digital epidemiology (Wolfe, 2011) processes to study population health and patterns of outbreak, as well as facilitate crucial contact tracing (Colizza et al., 2021). Murray and Piot (2021) emphasize the importance of aligning surveillance and public health response. Beyond this key role of state and local public health authorities, optimized longitudinal HRV monitoring combined with other remotely obtained data can be used in clinical practice as a highly sensitive vital sign indicating exacerbation of a patient's chronic condition or the onset of a new condition. While not currently available commercially, the rapid development of printed self-powered wearable sensors (Hai et al., 2021; Sempianatto et al., 2021) will further enable HRV related health innovations described in this proposal. Other technological innovations such as Shi et al.'s (2021) use of radar interferometry and LSTM networking eliminate the need for physical contact with the individual. Given the popularity of pursuing positive health and well-being, unobtrusive HRV monitoring can assist individuals in pursuing health maintenance and optimal performance goals. Beyond health and wellness applications, contributions to performance optimization in education, business and military/operational settings are feasible. But the immediate and, for some, vital imperative is to make the assessment and treatment of COVID 19 as accessible and useful as possible. Our project proposes longitudinal, wireless HRV monitoring to assist in the digital/algorithmic management and treatment of the COVID-19 pandemic in addition to other health conditions, using scalable, unobtrusive, and accurate technology.

## Author Contributions

RD and JT participated in the conceptualization and preparation of this opinion. All authors contributed to the article and approved the submitted version.

## Conflict of Interest

The authors declare that the research was conducted in the absence of any commercial or financial relationships that could be construed as a potential conflict of interest.

## Footnotes

^1^Statista.com/statistics/330695/number-of-smartphone-users-worldwide/.

## References

[B1] Ahmad S. Ramsay T. Huebsch L. Flanagan S. McDiarmid S. Batkin I.. (2009). Continuous multi-parameter heart rate variability analysis heralds onset of sepsis in adults. PLoS ONE 4:e6642. 10.1371/journal.pone.0006642 19680545 PMC2721415

[B2] Arias E. Tejada-Vera B. Ahmad F. (2021). Provisional Life Expectancy Estimates for January through June, 2020. Vital Statistics Rapid Release; no. 10. Hyattsville, MD: National Center for Health Statistics. 10.15620/cdc:100392

[B3] Chan A. T. Drew D. A. Nguyen L. H. Joshi A. D. Ma W. Guo C. G.. (2020). Harvard/Zoe COVID 19 Symptom Study. Available online at: https://covid.joinzoe.com/us/blog (accessed May 22, 2021).

[B4] Chung Y. T. (2020). Continuous temperature monitoring by a wearable device for early detection of febrile events in the SARS-CoV-2 outbreak in Taiwan, 2020. J. Microbiol. Immunol. Infect. 53, 503–504. 10.1016/j.jmii.2020.04.005 32331981 PMC7152863

[B5] Colizza V. Grill E. Mikolajczyk R. Cattuto C. Kucharski A. Riley S.. (2021). Time to evaluate COVID-19 contact-tracing apps. Nat. Med. 27, 361–362. 10.1038/s41591-021-01236-6 33589822

[B6] Dantzer R. Kelley K. W. (2007). Twenty years of research on cytokine-induced sickness behavior. Brain Behav. Immun. 21, 153–160. 10.1016/j.bbi.2006.09.006 17088043 PMC1850954

[B7] Denny J. Collins F. (2021). Precision medicine in 2030 -seven ways to transform healthcare. Cell. 184, 1415–1419. 10.1016/j.cell.2021.01.015 33740447 PMC9616629

[B8] Drury R. (2019). “HRV in an integrated hardware/software system using artificial intelligence to provide assessment, intervention and performance optimization,” in Autonomic Nervous System Monitoring, ed T. Aslanidis, 1–11. 10.5772/intechopen.89042

[B9] Drury R. L. Porges S. Thayer J. F. Ginsberg J. (eds.) (2019). Heart rate variability, health and well-being: a systems perspective. Front. Public Health 7:323. 10.3389/fpubh.2019.00323 31750285 PMC6848255

[B10] Fairchild D. (2013). Predictive monitoring for early detection of sepsis in neonatal ICU patients. Curr. Opin. Pediatr. 25, 172–179. 10.1097/MOP.0b013e32835e8fe6 23407184 PMC10989716

[B11] Grossman P. Taylor E. W. (2007). Toward understanding respiratory sinus arrhythmia: relations to cardiac vagal tone, evolution and biobehavioral functions. Biol. Psychol. 74, 263–285. 10.1016/j.biopsycho.2005.11.014 17081672

[B12] Hai L. Song H. Long M. Saeeda G. Lim S. (2021). Mortise–tenon joint structured hydrophobic surface-functionalized barium titanate/polyvinylidene fluoride nanocomposites for printed self-powered wearable sensors. Nanoscale 13, 2542–2555. 10.1039/D0NR07525F 33475650

[B13] Hasty F. García G. Dávila C. H. Wittels S. H. Hendricks S. Chong S. (2020). Heart rate variability as a possible predictive marker for acute inflammatory response in COVID-19 patients. Military Med. 186, e34–e38. 10.1093/milmed/usaa405 33206183 PMC7717314

[B14] Herry C. L. Cortes M. Wu H. T. Durosier L. D. Cao M. Burns P.. (2016). Temporal patterns in sheep fetal heart rate variability correlate to systemic cytokine inflammatory response: a methodological exploration of monitoring potential using complex signals bioinformatics. PLoS ONE 11:e0153515. 10.1371/journal.pone.015351 27100089 PMC4839772

[B15] Hirten R. P. Danieletto M. Tomalin L. Choi K. H. Zweig M. Golden E.. (2021). Use of Physiological Data From a Wearable Device to Identify SARS-CoV-2 Infection and Symptoms and Predict COVID-19 Diagnosis: Observational Study. J. Med. Inter. Res. 23:e26107. 10.2196/26107 33529156 PMC7901594

[B16] Holzman J. B. Bridgett D. J. (2017). Heart rate variability indices as bio-markers of top-down self-regulatory mechanisms: a meta-analytic review. Neurosci. Biobehav. Rev. 74(Pt A), 233–255. 10.1016/j.neubiorev.2016.12.032 28057463

[B17] Hood L. Dean K. R. Hammamieh R. Mellon S. H. Abu-Amara D. Flory J. D.. (2019). Multi-omic biomarker identification and validation for diagnosing warzone-related post-traumatic stress disorder. Mol. Psychiatry 25, 3337–3349. 10.1038/s41380-019-0496-z 31501510 PMC7714692

[B18] Jarczok M. N. Koenig J. Thayer J. F. (2021). Lower values of a novel index of Vagal-Neuroimmunomodulation are associated to higher all-cause mortality in two large general population samples with 18 year follow up. Sci Rep. 11:2554. 10.1038/s41598-021-82168-6 33510335 PMC7844270

[B19] Jarczok M. N. Koenig J. Wittling A. Fischer J. E. Thayer J. F. (2019). First evaluation of an index of low vagally mediated heart rate variability as a marker of health risks in human adults: proof of concept. J. Clin. Med. 8:E1940. 10.3390/jcm8111940 31717972 PMC6912519

[B20] Kovatchev B. P. Farhy L. S. Cao H. Griffin M. P. Lake D. E. Moorman J. R. (2003). Sample asymmetry analysis of heart rate characteristics with application to neonatal sepsis and systemic inflammatory response syndrome. Pediatr Res. 54, 892–898. 10.1203/01.PDR.0000088074.97781.4F 12930915

[B21] Leitzke M. Stefanovic D. Meyer J. J. Schimpf S. Schönknecht P. (2020). Autonomic balance determines the severity of COVID-19 courses. Bioelectron. Med. 6:22. 10.1186/s42234-020-00 33292846 PMC7683278

[B22] Malik M. Camm A. J. (2004). Dynamic Electrocardiology. New York, NY: Blackwell. 10.1093/oxfordjournals.eurheartj.a014868

[B23] McEwen B. (2017). Allostasis and the epigenetics of brain and body health over the life course. JAMA Psychiatry 74, 551–552. 10.1001/jamapsychiatry.2017.0270 28445556

[B24] Murray C. J. L. Piot P. (2021). The potential future of the COVID-19 pandemic. JAMA 325, 1249-1250. 10.1001/jama.2021.2828 33656519

[B25] Owens A. (2020). The role of heart rate variability in the future of remote digital biomarkers. Front. Neurosci. 14:582145. 10.3389/fnins.2020.582145 33281545 PMC7691243

[B26] Owens A. P. Friston K. J. Low D. A. Mathias C. J. Critchley H. D. (2018). Investigating the relationship between cardiac interoception and autonomic cardiac control using a predictive coding framework. Auton. Neurosci. Basic Clin. 210, 65–71. 10.1016/j.autneu.2018.01.001 29331491

[B27] Pavlov V. A. Chavan S. S. Tracey K. J. (2020). Bioelectronic medicine: from preclinical studies on the inflammatory reflex to new approaches in disease diagnosis and treatment. Cold Spring Harbor. Perspect. Med. 10:a034140. 10.1101/cshperspect.a034140 31138538 PMC7050582

[B28] Pavlov V. A. Tracey K. J. (2019). Bioelectronic medicine:updates, challenges and paths forward. Bioelectron. Med. 5:1. 10.1186/s42234-019-0018-y 32232092 PMC7098260

[B29] Perez M. V. Mahaffey K. W. Hedlin H. Rumsfeld J. S. Garcia A. Ferris T.. (2019). Large-scale assessment of a smartwatch to identify atrial fibrillation. N. Engl. J. Med. 381, 1909–1917. 10.1056/NEJMoa1901183 31722151 PMC8112605

[B30] Porges S. (2011). The Polyvagal Theory. New York, NY: Norton.

[B31] Rangon C. M. Krantic S. Moyse E. Fougère B. (2020). The vagal autonomic pathway of COVID-19 at the crossroad of Alzheimer's disease and aging: a review of knowledge. J. Alzheimers Dis. Rep. 4, 537–551. 10.3233/ADR-200273 33532701 PMC7835993

[B32] Sempianatto J. Lin M. Yin L. De la paz E. Pei K. Sonsa-ard T.. (2021). An epidural patch for the simultaneous monitoring of hemodynamic and metabolic biomarkers. Nat. Biomed. Eng. 172:112750 10.1038/s41551-021-00685-1 33589782

[B33] Shaffer F. Ginsberg J. (2019). An overview of heart rate variability metrics and norms. Front. Public Health 5:258. 10.3389/fpubh.2017.00258 29034226 PMC5624990

[B34] Shi K. Steigleder T. Schellenberger S. Michler F. Malessa A. Lurz F.. (2021). Contactless analysis of heart rate variability during cold pressor test. Sci Rep. 11:3025. 10.1038/s41598-021-81101-1 33542260 PMC7862409

[B35] Smarr B. L. Aschbacher K. Fisher S. M. Chowdhary A. Dilchert S. Puldon K.. (2020). Feasibility of continuous fever monitoring using wearable devices. Sci. Rep. 10:21640. 10.1038/s41598-020-78355-6 33318528 PMC7736301

[B36] Smith C. Gold J. Ngo T. D. Sumpter C. Free C. (2015). Mobile phone-based interventions for improving contraception use. Cochrane Database Syst. Rev. 2015:CD011159. 10.1002/14651858.CD011159.pub2 26115146 PMC6485989

[B37] Tarvainen M. P. Niskanen J. P. Lipponen J. A. Ranta-Aho P. O. Karjalaine P. A. (2014). Kubios HRV – heart rate variability analysis software. Comput. Methods Progr. Biomed. 113, 210–220. 10.1016/j.cmpb.2013.07.024 24054542

[B38] Thayer J. F. Fischer J. E. (2009). Heart rate variability, overnight urinary norepinephrine and C-reactive protein: evidence for the cholinergic anti-inflammatory pathway in healthy human adults. J. Intern. Med. 265, 439–447. 10.1111/j.1365-2796.2008.02023.x 19019182

[B39] Thayer J. F. Lane R. D. (2020). A model of neurovisceral integration in emotion regulation and dysregulation. J. Affect. Dis. 61, 201–216. 10.1016/S0165-0327(00)00338-4 11163422

[B40] Whitelaw S. Mamas M. A. Topol E. Van Spall H. G. C. (2020). Applications of digital technology in COVID-19 pandemic planning and response. Lancet Digit. Health 2, e435–e40. 10.1016/S2589-7500(20)30142-4 32835201 PMC7324092

[B41] Williams D. P. Koenig J. Carnevali L. Sgoifo A. Jarczok M. N. Sternberg E. M.. (2019). Heart rate variability and inflammation: a meta-analysis of human studies. Brain Behav. Immun. 80:219226. 10.1016/j.bbi.2019.03.009 30872091

[B42] Wolfe N. (2011). The Viral Storm. New York, NY: St. Martins.

